# Formation Techniques Used in Shape-Forming Microrobotic Systems with Multiple Microrobots: A Review

**DOI:** 10.3390/mi13111987

**Published:** 2022-11-16

**Authors:** Menaka Konara, Amith Mudugamuwa, Shanuka Dodampegama, Uditha Roshan, Ranjith Amarasinghe, Dzung Viet Dao

**Affiliations:** 1 Centre for Advanced Mechatronics Systems, University of Moratuwa, Katubedda 10400, Sri Lanka; 2 Department of Mechanical Engineering, University of Moratuwa, Katubedda 10400, Sri Lanka; 3 Queensland Micro- and Nanotechnology Centre (QMNC), Griffith University, Brisbane, QLD 4111, Australia

**Keywords:** multiple microrobots, formation techniques, assembly and disassembly, swarm robotics, collective actuation, selective actuation

## Abstract

Multiple robots are used in robotic applications to achieve tasks that are impossible to perform as individual robotic modules. At the microscale/nanoscale, controlling multiple robots is difficult due to the limitations of fabrication technologies and the availability of on-board controllers. This highlights the requirement of different approaches compared to macro systems for a group of microrobotic systems. Current microrobotic systems have the capability to form different configurations, either as a collectively actuated swarm or a selectively actuated group of agents. Magnetic, acoustic, electric, optical, and hybrid methods are reviewed under collective formation methods, and surface anchoring, heterogeneous design, and non-uniform control input are significant in the selective formation of microrobotic systems. In addition, actuation principles play an important role in designing microrobotic systems with multiple microrobots, and the various control systems are also reviewed because they affect the development of such systems at the microscale. Reconfigurability, self-adaptable motion, and enhanced imaging due to the aggregation of modules have shown potential applications specifically in the biomedical sector. This review presents the current state of shape formation using microrobots with regard to forming techniques, actuation principles, and control systems. Finally, the future developments of these systems are presented.

## 1. Introduction

Micro-/nano-electromechanical systems (MEMSs/NEMSs) are the technology related to the development of miniature devices with mechanical structures, sensing elements, and actuation components at the microscale/nanoscale. MEMSs comprise a multidisciplinary field that has rapidly evolved in recent years due to the major advantage of having a small footprint [1]. Advancements in MEMS technology have provided a positive impact on the development of technologies such as micromechanical [2,3], microthermal [4], micromagnetic [5,6], microoptical [7], microchemical [8], and most significantly, microfluidics, which use the behaviors of fluidic flows in micro-channels to perform various tasks [9,10,11]. In the biomedical sector, MEMS-based devices have demonstrated greater potential in disease diagnostics [12,13,14,15], detection and separation of bio-particles [16], and medical treatments [17]. Microrobots are a type of MEMS-based device with great potential in such applications [18]. Microrobotics is the technology related to designing, fabricating, actuating, and controlling miniature robots having characteristic dimensions on the microscale/nanoscale [19], and recently, it has emerged further with novel actuation principles [20], advanced micro-fabrication techniques [21], and the development of new materials [22].

With the development of MEMS devices, there has been a growing interest towards the design of controllable systems that can access enclosed smaller spaces such as inside the human body and microfluidic devices. As a result, microrobotic systems have been developed. Different actuation, feedback, and microfabrication techniques are used to implement these miniature systems. Noninvasive access, parallel operation, and opportunities to study micro-level physics/dynamics are significant advantages [23]. Microrobotic technology is widely investigated in the biomedical sector for in vivo and in vitro applications. In such applications, microrobots are designed to perform specific tasks such as delivering microparticles/nanoparticles, assembling or disassembling, cell manipulation, and sensing [24]. The main advantage of these robots is the capability to enter and navigate in microscale environments that are not accessible using macroscale robots or robotic tools [25]. Including these features in a microscale device has multiple challenges such as difficulties in fabricating and integrating complex components required for actuation and control, the presence of non-intuitive physical forces [23], and the limited applicability of remote field actuation methods [26]. Being MEMS devices, the design procedure and development techniques related to microrobots are also different from those used for macroscale robots [27,28]. Therefore, it is important to investigate specific microrobots to identify the potential of this technology and its applicability.

A group of robots that work together to achieve a common task has drawn the attention of the research community over the years due to their functionalities, which are impossible or inefficient to perform as individual robots. Such systems are called a swarm of robots, and at the macroscale, a wide range of applications such as collective exploration [29,30], pattern formation [31,32], and coordinated motion [33] are presented in the literature. The main advantages of robotic systems with multiple robots are adaptability, scalability, and robustness [34]. In considering robotic systems with multiple robots at the microscale/nanoscale, specific constraints have been identified in fabricating, controlling, and communicating [35]. Implementing actuators at the microscale/nanoscale is complicated due to limitations in fabrication. Furthermore, the smaller volume of the robots reduces the capability to integrate components required to actuate and control these robots. In comparison to the macroscale, the effects of forces are different at the microscale where viscosity and surface effects dominate over volumetric effects such as inertia and weight [36]. These limitations have led researchers to investigate microrobotic designs from a different perspective [37]. Available systems with multiple microrobots are controlled by external fields such as magnetic, acoustic, optical, and electric. Modules in these microsystems should have physical properties that make these systems responsive to actuation fields. This approach is different than controlling macroscopic robots via wireless signals where robots can receive control signals from remote controllers. Two types of control methods are identified from available microsystems. Several systems use multiple numbers of uniform modules and control modules as a single group, whereas others have focused on the selective actuation of individual robot modules. In these systems, the response of units to the actuation field is unique, allowing selective actuation. Planning, imaging, and controlling these systems require advanced control approaches [38]. Most commonly, research in this direction is focused on using these systems for biomedical applications.

In this review, microrobotic systems that consist of multiple robots are considered and the current state of shape formation using microrobots in such systems is presented. The collective and selective methods used to form shapes and assemble/disassemble are reviewed. Various actuation principles related to multi-agent microrobotic systems including magnetic, acoustic, electric, and optical actuation methods are available in the literature. The control systems used in these systems are also significant; therefore, the control techniques are reviewed in the later part of this work. Finally, an overview of the review is discussed in brief, and most importantly, the challenges and future directions of this research field are presented.

## 2. Shape Formation Techniques

In robotic applications, shape transformation using individual robots and shape formation using multiple robots provide the unique advantage of using the same device or the same set of devices having different shapes and configurations required to perform multiple operations. Shape formation using multiple agents is essential for applications such as the transport of therapeutic cargoes directly to target locations [39], manipulation of micro-objects [40], and rapid biosensing [41]. The significance is that the approach maximizes the navigation capabilities of the robotic system while eliminating the need to use various types of systems to perform multiple tasks related to a single application. Macroscale robots that form different configurations are either interconnected by active joints or actuated by responding to control signals, which guide them to form targeted shapes with the aid of inter-robot communications [42]. However, at the microscale, implementing intercommunication between robotic modules is not feasible due to limitations in integrating hardware components into small-scale robotic modules. Therefore, robots that form shapes are either assembled agent-to-agent individually or collectively acting under a global actuation signal without intercommunication between modules [43].

Formation techniques such as actuating microrobotic swarms as an individual system and selective actuation-based shape formation are reported with various types of actuation methods. Collectively, shape formation techniques and the actuation methods offer various capabilities and also limitations to the microrobotic system, which significantly affect the functionality and the performance when dealing with multiple microrobots. Key features of different shape forming techniques are shown in Table 1.

### 2.1. Shape Formation by Microrobotic Swarms

Microrobots that are actuated as a group using an external energy field without a physical connection between them are commonly reported. Microscale and nanoscale particles are investigated towards the formation of various shapes. Particle-scale microrobots are able to form complex shapes such as chains, vortexes, and ribbons [64]. In most cases, micro-agents in these systems are uniform and smaller. Further, they cannot be actuated individually, mainly due to their smaller modular size, which makes it complex to integrate fabrication differences, use anchoring methods, and applying non-uniform control inputs. However, such approaches are commonly used in selectively actuated reconfigurable microrobotic systems.

#### 2.1.1. Magnetic Formation

**Figure 1:**
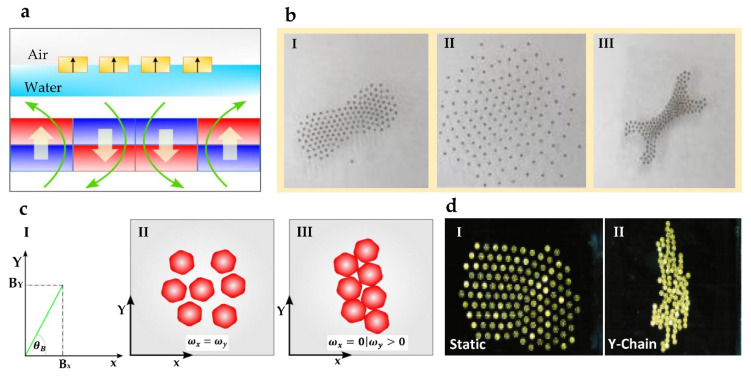
Shape formation techniques using magnetic fields: (**a**) Side view of programmable magnetic array that is used to change the resultant magnetic field on the air–water interface. (**b**) Representative example of microparticles aggregating to form the target formation under the resultant magnetic field generated by the magnetic array. Reproduced with permission from [40]; published by SAGE Publications. (**c**) The graph represents the resulting magnetic field from two orthogonal magnetic fields. The two figures show how microrobots form different formations when the applied magnetic field frequencies ($\omega_{x},\;\omega_{y}$) are equal to or greater than the other. (**d**) Representative example of micro-disk formations into static (when $\omega_{x}=\omega_{y}$) and Y-chain formations (when $\omega_{x}=0;\omega_{y}>10$) with magnetic field frequency variation. Reproduced with permission from [67]; published by Springer Nature.

Particle formation using an external magnetic field has been reported with various types of microparticles with magnetic properties. In most applications, hard materials (NdFeB, FePt, Alnico, SmCo, Cr, and Cro2) and soft materials (Fe_3_O_4_/SPIONs, Ni, Co, Gd, NiFe, and FePt) are used [52]. During formation, particle–particle forces and particle–external field forces affect the motion of agents [65]. Programming external ferromagnetic arrays [40] and changing the magnetic field frequency [45] and orientation [66] are several methods discussed in the literature to form configurations using microparticles. The magnetic field variation of a ferromagnetic array as shown in Figure 1a manipulates particles into different static formations and navigates particles as a swarm [40]. Forming target static shapes such as letters requires encoding energy maps and manufacturing an external magnetic array based on the target shape. The formation of a target shape under the applied magnetic field is shown in Figure 1b. Changing the magnetic field orientation and the distance between particles and magnets reconfigures the microparticle formations. Alternating magnetic fields are used with hematite colloidal particles to form liquid, chain, vortex, and ribbon-like microrobotic swarms, and the particles interchange between the formations to perform different tasks [45].

Changing the frequency and polarization of the magnetic field in 3D has been used to control microparticles in many studies using magnetic field combinations that are orthogonal to each other, as shown in Figure 1c [68]. The behavior of the formation changes with each magnetic field’s frequency, where different modes such as static and linear formations (as shown in Figure 1c) are formed. The formation of different shapes is achieved as a result of a magnetic dipole, hydrodynamic thrust, and repulsive forces acting on microparticles [45]. In a magnetic microdisk system, the collective is configured into six different formations (rotation, oscillation, static, chains, oscillating chains, and gas-like mode containing self-propelling pairs) by altering the external magnetic field frequency [67]. Depending on the magnetic field frequency, the microdisk collective interchange between static and Y-chain formation as shown in Figure 1d. When corrugations around microdisks are aligned, the robots are attracted, whereas an angular misalignment can result in the repulsion of the formation. In the same study, various functions such as channel crossing, splitting, adapting to the environment, and object rotation have been successfully demonstrated. Self-assemble chain swarms of supermagnetic microparticles are generated by magnetic guidance systems, and morphological changes are obtained by changing the precision angle and tilt angle of the magnetic field [66]. Self-organizing behavior is important in using magnetically actuated microparticles for biomedical applications such as imaging and targeted drug delivery [45]. In biomedical applications, magnetic actuation-based manipulation provides advantages such as minimal interaction with tissues and transparency in detecting the particles in the human body [64]. However, managing the field strength is important when used for biomedical applications.

#### 2.1.2. Acoustic Formation

The creation of pressure nodes or anti-nodes is used to gather microparticles in acoustic-based actuation. Specific microparticle materials are not needed in the acoustic method whereas magnetic and electric actuation methods, respectively require magnetic and dielectric materials [69]. Further, differently sized microparticles ranging from 10^−7^ m to 10^−2^ m are used with this method, due to the wide acoustic wave frequency range [44]. Acoustic actuation for forming different configurations has the capability to control formations deep inside the human body, with higher flexibility and lower power consumption. Acoustic tweezers [70], acoustic transducers [71], and travelling-wave (TW)-based methods [72] have been used to generate formations using microparticles. Acoustic tweezer systems form shapes and transport larger objects with the help of pressure fields by altering the position, shape, and number of tweezers [47]. Further, morphological transformations such as reversible elongations and splitting from one swarm to multiple, and merging back are performed. Any granular material can be used in this system to form swarms, and the formations have better environmental adaptability [47].

**Figure 2:**
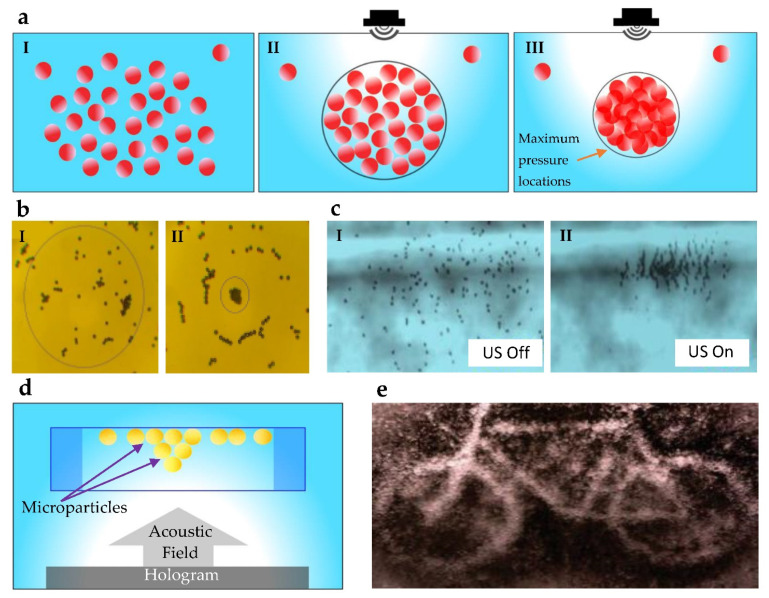
Shape formation techniques using acoustic fields: (**a**) Under an applied acoustic field, particles move towards minimum pressure locations. Circle diameter variation shows the convergence of pressure maxima on the water surface. (**b**) Acoustic traps generate the formation of microparticles by creating pressure minima surrounded by regions of a high pressure amplitude. Reproduced with permission from [69]; published by AIP Publishing. (**c**) Representative example of the rapid-ultrasound-triggered assembly of nanomotors into a swarm. Reproduced with permission from [48]; published by American Chemical Society. (**d**) An acoustic field is applied through the hologram to make target assemblies. (**e**) An arbitrary shape produced using travelling waves (TW) and holograms. Reproduced with permission from [75]; published by John Wiley and Sons.

Microparticles that are spread over a plane are brought together by progressively generating traps, as shown in Figure 2a, with acoustic tweezers, and it shows the capability of the method, although the method is relatively simple [69]. The generation of acoustic traps was carried out by a circular ultrasonic array, varying the phase and amplitude of the signal. Such a creation of an acoustic trap is shown in Figure 2b. The motion of a reversible assembly of nanomotors is externally controlled using either frequency or voltage by acoustic transducers [48]. Nanomotors assemble in the presence of an acoustic field, as shown in Figure 2c. In creating 3D pressure fields, asymmetric microparticle swarms are formed using the travelling wave (TW) method [73]. PDMS spheres on the water are used as microparticles, and the system has a higher degree of freedom than conventional phased array transducers. Hologram setups similar to Figure 2d are used to develop arbitrary shapes using the TW method. A complex shape assembled using a hologram approach is shown in Figure 2e. The ability of the acoustic method to control the collective behavior of nanomotors is important for future applications such as nanomachines that function collectively and mimic animal swarms [48]. These nanomotors can be used in the fields of nanomedicine, nanofabrication, and cargo transport. In those applications, the biocompatibility of acoustic actuation is vital. However, proper instrumentation is required for applications such as in vivo experiments [74].

#### 2.1.3. Electric Formation

**Figure 3:**
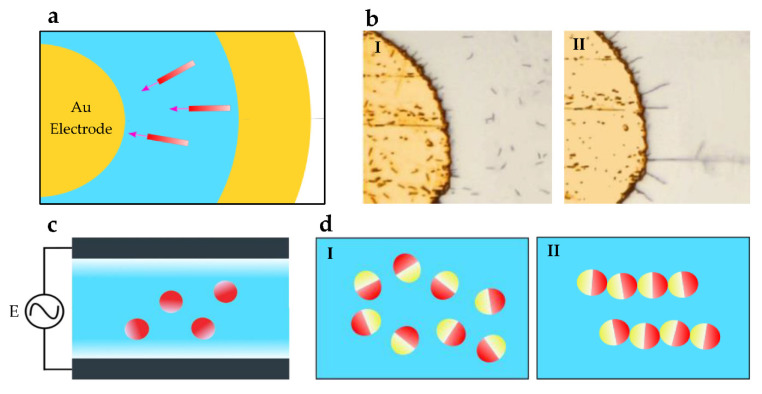
Shape formation techniques using electric fields: (**a**) Circular electrode nanowire actuation setup. (**b**) Representative example of randomly distributed nanowires aligning radially under the application of an electric field. Reprinted from [51], Copyright (2011), with permission from Elsevier. (**c**) An AC electric actuation experimental setup with top and bottom electrodes. (**d**) Colloids that have metallic and dielectric hemispheres form swarms and chains with varying external frequencies [49].

The motion of microparticles under electric actuation occurs as a result of interaction forces between dielectric particles and the electric field. Polarized particles move towards electrodes having opposite polarization under Coulomb interaction. This is identified as the dielectrophoretic (DEP) effect [44]. DEP torque and electrophoretic force (EP) have been used to manipulate nanowires, with electric tweezer systems [51]. These nanowires are higher aspect ratio modules, and when they reach equipotential lines under an electric field, various swarm configurations are formed. Electrode setups similar to Figure 3a have been used to form nanowire arrays, as shown in Figure 3b. Electric tweezers use DC and AC electric fields to precisely assemble nanowires into different collective formations in liquids. In these systems, oppositely charged nanowires are combined regardless of their initial large separations. Propulsion is achieved using EP forces, whereas DEP torque is responsible for the directional control of the motion. Colloids are microscopically spread insoluble nanoparticles used to form swarms [76]. Actuation setups similar to Figure 3c have been used to generate EP forces on colloids in fluidic environments. Colloids that are fabricated as silica spheres having one hemisphere coated with metal have been reconfigured into various collective states, as shown in Figure 3d, using AC electric field interactions [49]. The polarization difference between the two hemispheres leads to imbalanced electrostatics and self-propulsion. The dielectric response of these colloids depends on the external electric field frequency. The change of frequency forms coherent swarms, and active chains are produced by changing dipolar interactions.

Another method is to generate electrohydrodynamic (EHD) flows using charges induced on the electrodes [77]. The spatial current distribution in the electrodes is a critical factor in using this method [78]. EHD flows are used for swarm generation. Microparticles with different dielectric properties and sizes are assembled and moved as a swarm using EHD [50]. The converging EHD flow increases the interactions between particles and forms a hierarchical swarm. Furthermore, swarms also move due to EHD flow, and more particles join the group as it moves through the fluid. The speed of the formation varies with the applied voltage, frequency, and the number of particles. Potential application areas of the electric actuation-based swarm formation technique are cell-specific drug delivery, the development of nanowire motors, oscillators, and intelligent microrobot/nanorobot systems for biomedicine and microengineering [50,51]. A significant challenge in this method is applying electric fields to actuate microrobots for in vivo applications [65].

#### 2.1.4. Optical Formation

When microparticles are actuated using optical methods, they absorb energy from an external optical source. This energy is converted to temperature gradients in the fluid, which guide the microparticles. In other instances, heat initiates chemical reactions in the fluid and creates a chemical gradient field that affects the motion of the particles [44]. These two methods are identified as photothermal and photochemical methods. In photothermal approaches, the evanescent-field-based micromanipulation method is used for particle trapping and moving. In general, these investigations are conducted using polystyrene particles and red blood cells [79]. Microparticles have been guided as a macroscopic ensemble consisting of thousands of particles. The speed of particles is controlled by adjusting the laser power. The self-thermophoretic motion of particles has been used to form swarms using photothermal actuation [80].

**Figure 4:**
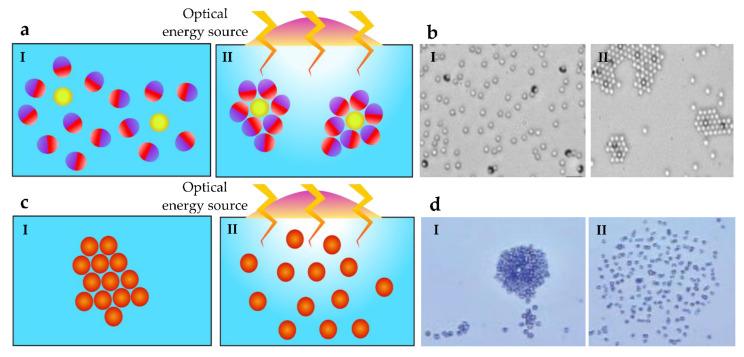
Shape formation techniques using optical methods: (**a**) In the presence of a light field, microparticles aggregate as a photochemical reaction. When the field is removed, particles move away from each other. (**b**) Representative example of TiO_2_ active particles forming clusters under UV actuation. Reversible collectives disperse as a result of Brownian diffusion. Reproduced with permission from [54]; published by John Wiley and Sons. (**c**) In the absence of a light field, microparticles aggregate; however, when a light field is applied, particles disperse from each other. (**d**) Representative example of colloids gathering around TiO_2_ active particles. Neighboring particles move away from each other under UV actuation. Reproduced with permission from [83]; published by John Wiley and Sons.

A comet-like swarm is generated by optically driving colloids under self-thermophoretic motion [53]. The light energy received by each colloid results in increasing the temperature of the colloids and the fluid. Further, the asymmetric temperature distribution in the top and lower hemispheres of a colloid could lead to self-propulsion through self-thermophoresis [81]. In photochemical approaches, under external light fields, particles assemble into formations, as shown in Figure 4a. Particles that are composed of SiO_2_ and TiO_2_ are propelled in the presence of UV light, as shown in Figure 4b [54]. Then, clusters are formed using inactive silica particles. The capability to self-assemble with passive objects has been shown by AgCl micromotors under UV actuation [82]. The motion of these particles occurs due to asymmetric photodecomposition. In some studies, microparticle assemblies disperse under UV actuation, as illustrated in Figure 4c. Colloids that are gathered around TiO_2_ particles move away from each other (as shown in Figure 4d) when an optical source is applied as a result of multiple mechanisms [83]. The incapability to penetrate deeply is a challenge in using optical actuation, but it is suitable for biotechnology, lab-on-a-chip, and organ-on-a-chip applications where deep penetration is not required. Optical manipulation can be used for long-range applications and have advantages in energy efficiency and precise actuation [52].

#### 2.1.5. Hybrid Methods

Hybrid concepts have the potential to integrate the advantages of different actuation methods. In these systems, microrobots have the capability to respond to different types of actuation fields. This behavior is either integrated as a material property [84] or as design features that respond to both actuation fields [85]. In the former method, particles have material properties such as supermagnetic, paramagnetic, and dielectric and respond to external fields. For hybrid actuation, other material-independent actuation fields such as acoustic and optic are used.

**Figure 5:**
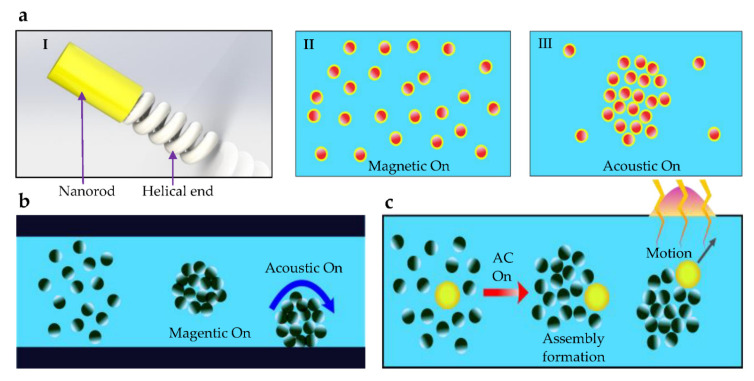
Shape formation techniques using hybrid methods: (**a**) Schematic illustration of the design of magneto-acoustic nanomotors and reversible assembly formation [84]. Under a magnetic field, swarm motion is achieved, whereas the application of the acoustic signal forces the nanomotors to aggregate. (**b**) Supermagnetic particles aggregate as a result of an external magnetic field. When the acoustic field is applied, the collective rotates along the surface [85]. (**c**) Under electric actuation, TiO_2_ particles assemble into a formation, and the UV field allows precise motion control of the swarm along the required trajectory [50].

Designing microrobots to have two separate features and components that can respond to different fields is used in the design feature approach. Different swarm motions such as directional motion as a group and assembly and disassembly functionalities have been demonstrated using magnetic–acoustic hybrid nanomotors [84]. These nanomotors are molecular machines that can convert energy into physical movement [86]. Microrobots with helical and concave rod swimmer designs (as shown in Figure 5a) have utilized both magnetic and acoustic propulsion. Changing the actuation field direction and switching between two actuation methods are used to manipulate nanomotors to aggregation and dispersion, as shown in Figure 5a. Supermagnetic and paramagnetic particles have been used with hybrid actuation to create formations. External magnetic and acoustic fields are used to aggregate and produce rolling-type motions using supermagnetic particles, as shown in Figure 5b [85]. Aggregation and dissolution are performed by the magnetic field, whereas rolling motion is carried out by applying the acoustic field.

Paramagnetic nanoparticles are formed into tornado-like swarming patterns using both magnetic and optical actuation methods [87]. Planar actuation of the swarm is performed by a magnetic field, whereas vertical motions are carried out by optical actuation. The directional motion of an electrically assembled TiO2 particle swarm has been controlled using UV light, as shown in Figure 5c [50]. EHD-flow-based motion control is less effective, and the use of the optical method has allowed proper navigation of a leader– follower-like swarm [44]. Hybrid actuated robots provide the ability to integrate imaging and diagnostic capability, which is specifically advantageous in biomedical applications [84]. The capability to roll through microfluidic channels demonstrates the potential in nano-drug delivery to guide toward hard-to-reach capillaries, which are identified as a potential application of hybrid actuated supermagnetic particles [85].

### 2.2. Shape Formation by Selectively Controlled Multiple Microagents

Selective control of agents is the other method used to form shapes in microrobotic systems and work as a group. In contrast to the collective actuation of swarms, which are basically microparticles/nanoparticles, these selective systems are relatively larger in individual module size. The larger module size allows the implementation of selective control techniques and related hardware to individual microrobots. Several approaches to achieving selective control are surface anchoring [55], introducing different geometrical features in fabrication [58], and applying non-uniform control input methods [55].

#### 2.2.1. Surface Anchoring

In these types of systems, microrobots work in specifically designed control surfaces and the modules are selectively anchored to the surface to form shapes. This method is commonly applied to magnetic microrobots using electrostatic anchoring [56]. Magnetic microrobots are selectively actuated using electrostatic control surfaces built with interdigitated electrodes. The activation is performed by applying different anchoring voltages to the interdigitated electrodes on the surface. The anchoring voltage value depends on the current velocity of the module. When required, individual module motion is stopped by activating surface electrodes. In these systems, microrobots are actuated as a group using external magnetic fields, and unanchored modules continue their motion under the effect of that external magnetic field. Frictional and adhesive forces are significant in defining the motion of unanchored devices [55].

**Figure 6:**
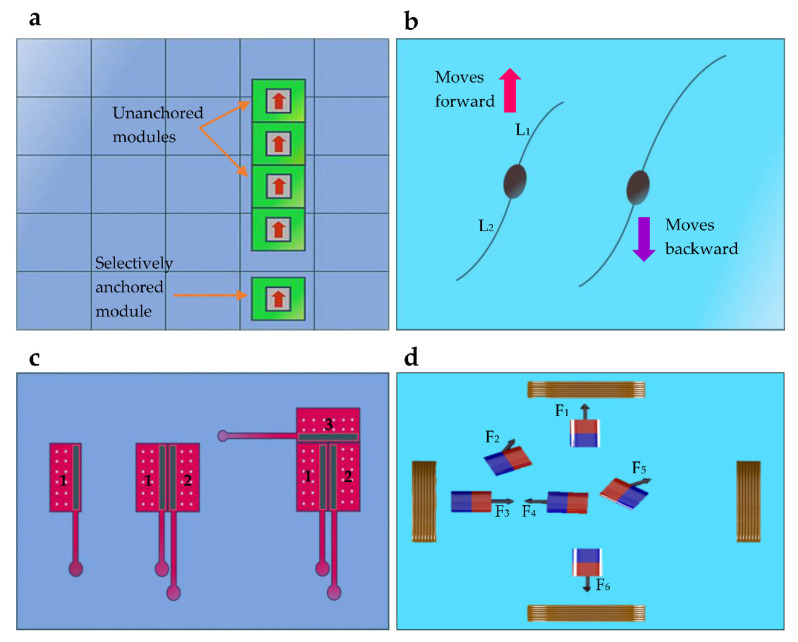
Shape formation techniques using selective control methods: (**a**) In the surface anchoring method, some modules are fixed to the surface selectively, whereas other modules navigate under external actuation [55]. (**b**) A two-tailed swimming microrobot design, where, depending on the tail length ratio (l1l2), the modules behave differently [59]. (**c**) Modules with differently sized arm-type structures exhibit different motions under the same actuation signals. These modules are navigated to form different shapes [88]. (**d**) Due to the spatial variation of the magnetic field, different microrobots experience different resultant forces [89].

Electrostatic anchoring has been used for assembling and disassembling magnetic microrobots into different configurations by anchoring modules, as shown in Figure 6a. Several modules can be fixed at specific locations by localized anchoring. Then, the other modules are moved towards the static modules during the assembly process. When the modules are sufficiently close, magnetic forces between static and moving modules cause them to combine [35]. When the interconnection forces are stronger, changing to different shapes requires higher disassembly forces; therefore, an outer shell can be added around the modules to reduce the strength of the interconnection [57]. Module interconnection has been avoided by maintaining a minimum distance between modules in several studies, but doing so eliminates the ability to form different shapes using interconnectivity [55].

#### 2.2.2. Heterogeneous Design

In these systems, modules behave differently under the same global actuation signals. Most commonly, this is implemented by making geometrical variations in the modules. The change of internal magnetization has also been used to selectively control the modules. A geometrical variation has been used to selectively actuate two-tailed swimming microrobots [59]. Depending on the magnetic field frequency, the propulsion forces generated by the two tails are varied. This allows controlling individual microrobot motion between forward and backward directions, as shown in Figure 6b. At the reversal frequency, microswimmers obtain a zero swimming speed [59]. Various micro assemblies are formed using stress-engineered modules and specifically designed control platforms [58]. Modules have an arm-type structure that responds differently to voltage signals based on the dimensions of the arm. These steering arm actuators have different transition voltages, which can raise and lower the arms. Modules with different transition voltages are individually controlled, and forward and turning motions can be performed using the pre-defined transition voltage. Control signals are transmitted through interdigitated electrodes on the surface. Comparatively, this approach proposes a force closure concept to form target shapes that are similar to the formation shown in Figure 6c. Modules are not interconnected, but form configurations as a result of force equilibrium [88]. Each robot is capable of connecting with the others, and shape forming is planned through algorithms [90].

Geometrically different magnetic microrobots are fabricated and have demonstrated different responses to the same actuating magnetic field [91]. When microrobots are different in size, their rotational inertias are also different. This results in distinct vibrational responses under the magnetic actuation field. Microrobots that are larger in length have higher rotational inertia, reach lower angular accelerations, and have smaller total angular swings [60]. Extending this method to multiple robots requires reduced coupling between the modules. The change of shape demagnetization factor has been used to design unique soft magnetic microrobots. When the aspect ratios of modules are different, this results in a variety of shape demagnetization [92]. Constant cross-section and constant length approaches are used to introduce different aspect ratios. The variation of shape demagnetization affects the internal magnetization of the modules [60]. Due to that, the modules behave differently under the same magnetic actuation field. The use of distinct critical frequencies of magnetically actuated propellers for selective control of microrobots has been reported [93]. When microrobots have distinct critical frequencies, their speed–frequency relationships are different. Actuating them below or above their critical frequencies is used for individual steering. Internal magnetization of hard magnetic microrobots has been altered to change the resultant magnetic torques. Due to that, a module behaves differently. The module with the highest magnetization moves in many operating conditions, whereas the modules with lower magnetizations move in limited conditions [60]. Different levels of magnetic field strength and oscillation frequency only allow selective movements of the modules, and the desired configurations are formed. Microrobots with different magnetizing strengths (N42 and N52 grade) have been used to demonstrate motion differences under the same actuation fields. The forward velocity of the N42-grade is slightly higher than that of the N52-grade module under the same rotating frequencies [94]. Further, magnetic heterogeneity is used in microrobots developed with various bead types to exhibit different velocities and moving directions under the same control input [95].

#### 2.2.3. Non-Uniform Control Input

The change of the control input acting on different modules has been used to independently control identical and non-identical magnetic microrobots. Forces acting on the modules are different between each other and selectively controllable. In these studies, microrobots are kept sufficiently apart from each other to avoid the effect of interactive force on the motion. This separation has further allowed for the introduction of distinct forces on modules using spatially varying external magnetic fields [61,62]. The superposition of the magnetic fields is used to manipulate microrobots in different trajectories under non-uniform forces acting on each module [89], as shown in Figure 6d. Microrobots are moved in the same and opposite directions. Generating forces in opposite directions has been identified as a complex task when the modules are close to each other. The use of non-uniform control inputs for the selective control of modules has shown more potential for applications, as it does not need precise microfabrication and can be used with both identical and nonidentical modules [63].

#### 2.2.4. Other Methods

**Figure 7:**
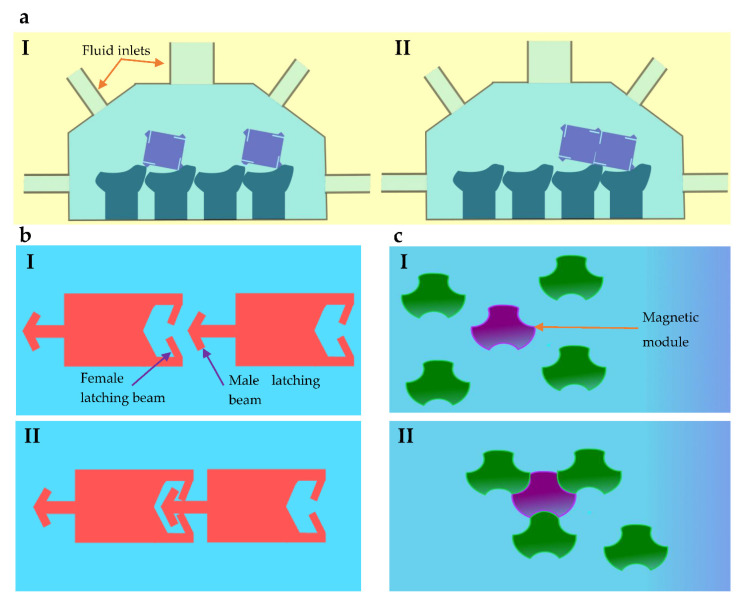
Other selective control methods: (**a**) Silicon tile assembly formation using selectively controlled fluidic flows [96]. (**b**) Micromodules that have male and female connecting structures are interconnected using a rail-guided method [97]. (**c**) Magnetic modules are guided selectively to bond connections with other non-magnetic modules using thermal bonding [98].

Some systems have used different methods for selective control other than the most common approaches discussed in previous sections. Latch mechanisms have been used to connect modules with one another, as shown in Figure 7a [96]. This approach uses fluid flows to selectively guide individual components. The study has demonstrated linear row, pair, and L-shaped assemblies where fluid flow valve sequences define the final output shape. Further, the chamber of the system is designed with a geometric pattern to align modules for the bonding procedure. The method has provided larger flexibility when it comes to output shapes, but the drawback is the requirement of advanced controllers for flow actuation.

Railed microfluidic channels are used to guide and assemble microstructures individually [97]. In this method, each module consists of a male latching beam in front and female beams on the other end, as shown in Figure 7b. The male latching beam of one module deflects the female beams of another, forming an assembly under a fluid flow motion. These bonds are irreversible, and the assembly moves together even after the flow is reversed. In another method, modules are selectively aggregated into a formation one-by-one. A single magnetic module is manipulated to pick other non-magnetic modules. The modules consist of thermal bonding faces around the outer surface of the body. When the modules are closer to the magnetic module, the temperature is increased, allowing bonds to form, as shown in Figure 7c. Two methods are used for heat-activated bonds as global conduction through the surrounding liquid and localized heating through a focused laser [98].

### 2.3. Advantages and Challenges of Different Techniques

Different techniques have been used to create formations using microrobots. The methods discussed in the previous sections have shown clear distinct approaches toward shape formation. As an emerging study area, it is important to identify the advantages and limitations of each method so that an evaluation can be carried out to select the best method for a specific application. In Table 2, the identified advantages and challenges are summarized.

## 3. Control Systems

**Figure 8:**
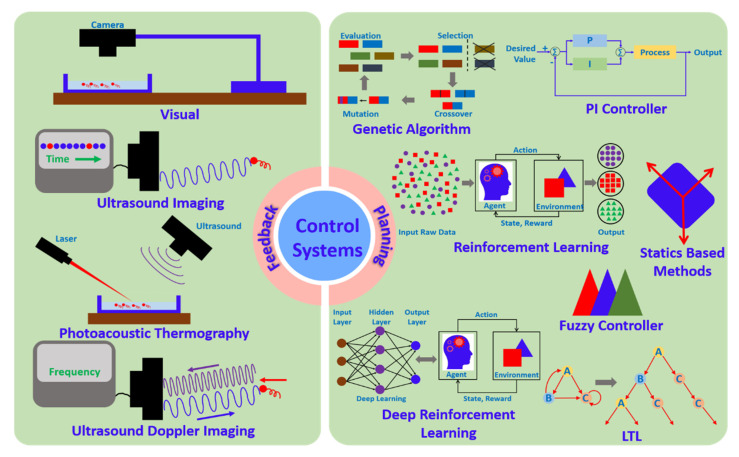
Schematic diagram showing different feedback and planning methods.

In order to achieve target shapes using multiple microrobots, several systems have used feedback systems. Closed-loop controllers are required to improve the accuracy of long-term operations. Further, algorithms are used to find optimized steps to form target shapes. Planning and feedback control methods used in shape forming systems are shown in Figure 8. Optimization of self-assembling magnetic droplets has been carried out using real-time visual feedback and a genetic algorithm (GA) [104]. A magnetized needle attracts all particles to form an assembled pattern, and the path of the needle is planned using a hybrid genetic algorithm [105]. The optimized path is the shortest in length and connects all droplets. Snakelike magnetic swarms are generated and optimized with the help of genetic algorithms. The generation process has been considered as an open-loop travelling salesman problem [106]. Starting with a single microrobot connecting with another, but slower microrobot, snakelike formations are generated in these studies. The shortest tour generated by the GA traverses all microrobots. A reinforcement learning control scheme has been proposed for the flow navigation of smart micro-swimmers [107]. Numerical experiments have shown that swimmers learn optimal strategies by experience. For three types of microrobots having a different degree of freedom (DoF), a reinforcement-learning-based control scheme for navigation in a free environment and an obstacle environment was proposed and validated [108]. The deep reinforcement learning algorithm has the capability to use with experimental systems as it can directly process raw sensor inputs. Planning algorithms have been used for stress-engineered micro-assemblies, and control strategies are implemented using an iterative re-planning algorithm [58].

For shape deformation control of mobile paramagnetic nanoparticle swarms (EPNSs) fuzzy-logic-based control schemes have been used. Considering issues that can occur with a conventional PI controller such as large overshoot and instability due to long-time shape deformation, two fuzzy gain controllers for K_p_ and K_i_ of the feedback controller are implemented [109]. Stable automatic control of shape deformation has been achieved during translational and rotational motions. Some studies have used PI controllers in order to find the required orientations of magnetic coils to selectively control multiple microrobots [61]. For automated control of paramagnetic nanoparticles, statics-based methods are used [110]. Swarm distribution, morphology, and location are described using the statistical parameters such as the number of particle units and total distributed area. This allows handling swarm behavior quantitatively afterwards. Some studies have used linear temporal logic (LTL) control frameworks for planning micromanipulation tasks using magnetic microrobots [111].

Ultrasound imaging feedback is used for real-time magnetic navigation of rotating colloidal microswarms for in vivo applications [112]. Experiments have been conducted at various depths for the localization of these swarms. Ultrasound imaging has provided fast imaging speeds [113]. The visual servoing method has been proposed to control swimming microrobots [114]. The vision module with a stereo camera in the system obtains the location of microrobots, while the motor module actuates a delta mechanism to control the vertical position of the camera. Ultrasound Doppler imaging guidance is another feedback method used for magnetic microswarm navigation, especially in endovascular delivery [115]. The rotating microswarms are tracked in real-time in stagnant and flowing blood conditions. Photoacoustic computed thermography (PACT) has been used for real-time visualization of a microrobotic system for targeted navigation in intestines in vivo [116]. This allows precise control of microrobots using deep imaging for targeted drug delivery. Ultrasonic and photoacoustic imaging techniques are used to track liquid metal nanobots in vivo and in vitro [117]. Further, magnetic resonance imaging (MRI) is used to track magnetotactic bacteria (MTB) nanorobots in 3D inside the human body [118]. These nanobots are used for targeting specific locations in microvasculature where the tracking is necessary. Vision feedback has been used in formation controllers to find the positions of microrobots [119]. In this approach, the leader trajectory has been used as the reference for follower microrobots.

## 4. Discussion and Future Directions

The use of multiple microrobots has seen growing interest in recent years mostly towards biomedical applications. Such systems are developed in various forms to perform complex tasks that are difficult to carry out using conventional medical robotic systems or individual microrobots. The significance is that, although animal trials have been conducted for multiple microrobotic systems, it has been identified that there is a lack of investigations that extend to human trials. Primarily, multiple microrobotic systems are categorized as collectively actuated and selectively actuated systems depending on the controllability of the microrobotic agents. Collectively actuated swarms are controlled as a group using external actuation fields. On the other hand, selectively controlled agents are also of interest depending on the application. The use of a higher number of modules, which is commonly seen in collectively actuated systems, has shown promising potential in internally diagnosing and treating the human body because multiple microrobots have a comparative advantage in tracking agents when imaging is used. Further, a higher drug delivery capacity can be achieved during treatments. The proper identification of the design requirements of micromodules is important to improve the applicability of a single microrobotic system in multiple applications. Specific applications such as clot removal in blood vessels, medical cargo delivery, and targeted therapy need specific designs. Several microrobotic systems are designed based on different physical properties such as the critical frequency of swimmers, the magnetization strength of magnetic modules, and the lengths of structural components to perform selective control. In such systems, increasing the number of modules and obtaining precise directional control require further design improvements.

Magnetic, acoustic, electric, and optic actuation are the most commonly used techniques in systems with multiple microrobots. In addition, hybrid methods are used for collective actuation. Magnetically actuated systems rely on magnetic torque and gradient- based methods to control the modules. In contrast with other actuation techniques, variations of the external field frequency and orientation have allowed forming a wide range of collective configurations. Additionally, the capability to control the magnetic field precisely has been significant even for selectively controlled systems. Electrically actuated systems require dielectric particles to be manipulated using AC and DC electric fields. The created formations have shown fast responses to external stimulations. With respect to acoustic systems, pressure gradients are used to form swarms, and this reduces the particle loss during the formation process. In addition, specific materials are not required when compared with magnetic and electric actuation-based systems. However, acoustic wave generation highly relies on the working environment. With the use of optical-energy-based systems, spatially selective formations can be achieved using relatively simpler actuation setups. However, in the biomedical sector, the applicability of this method has limitations due to the lower penetration depth. Hybrid actuation methods have been developed to overcome the limitations in using a single actuation technique. The combination of two actuation methods allows utilizing key features of both techniques in one system. Electro-optic actuation is significant where rapid formation under an electric field and precise motion control under an optical actuation are combined.

Mainly, selective control is introduced using surface anchoring, heterogeneous designs, and non-uniform control input methods. In comparison, surface anchoring is a more straightforward technique to selectively fix and move modules to form target configurations. Heterogeneous designs are developed based on different geometrical and physical properties. Investigations towards stress-engineered microrobot development have initiated new research areas on untethered microrobot controlling. Specifically designed control surfaces or modules with different physical properties are not required when the non-uniform input method is used. However, applying non-uniform control forces on the modules is computationally expensive. Further, directional control is complex when the modules are closer to each other. Shape forming with acoustic holograms and fluidic assemblies has shown potential applicability in the microfabrication of devices. For example, microscale modules of different materials that are integrated with sensors and actuators can be assembled into a single device. Further, the acoustic assembly provides a rapid method to fabricate in parallel arbitrary 2D shapes using holograms.

The applicability of these systems in clinical applications has challenges in terms of precise locomotion, accurate feedback control, and adaptability to dynamic environments. Therefore, most investigations are limited to in vitro or ex vivo setups, which are only able to prove the concepts. The 2D planar motions are generally investigated, but in in vivo environments, precise manipulation in complex 3D environments is required. At present, the aforementioned actuation techniques lack reliable kinematic and dynamic models, which restricts the applicability to real scenarios. Being a favorable candidate for biomedical applications, microrobotic systems rely on these biocompatible actuation methods such as magnetic- and acoustic-actuation-based systems. The biocompatibility of multiple microrobotic systems is an essential parameter for real-world medical applications, which require interactions with living cells. Bio-hybrid agents and particles enclosed in plasma and cell membranes increase the biocompatibility of these systems. After treatments or diagnostics, microrobots should either be removed or they should be degradable. Furthermore, they should not be toxic to the human body, even after mixing with body fluids. For example, materials such as nickel and cobalt are widely used in developing microrobots, but direct contact with human tissues is avoided to ensure safety by coating individual microrobots with non-toxic materials. Hydrogel-based microrobots/nanorobots are used to reduce the toxicity of swarms.

Controllable microrobots are able to perform multiple functionalities such as navigation through narrow channels and delivering drugs to targeted locations. Existing systems use feedback for the localization and navigation of multiple modules. Precise feedback is important in controlling, yet challenging in biomedical applications, mainly because the treatment procedures are carried out in a highly dynamic in vivo environment, which generates various noises in the feedback signals. Intelligent controllers are widely used in macroscale swarms for precise feedback controlling and motion planning. Limitations in fabrication techniques have reduced the ability to apply the same control methods and systems at the microscale/nanoscale. However, several studies have demonstrated the applicability of intelligent controllers for multiple microrobotic systems. With a higher number of modules, the importance of advanced intelligent controllers is highlighted. The integration of intelligent controllers with machine learning, GA, and evolutionary strategies are identified to be useful in finding optimal solutions for controlling multiple microrobots. Targeted navigation and obstacle avoidance of microswarms is achieved with the use of path planning algorithms such as iterative re-planning and travelling salesman problem algorithms. For specific applications such as image-guided therapy and minimally invasive surgery, the involvement of medical imaging systems is required. Further, multimodal imaging that combines the advantages of several technologies such as MRI and single-photon emission computerized tomography can be beneficial for clinical applications.

Multiple microrobot systems use different techniques to create formations at the microscale. Based on controllability, these techniques can further be classified as collectively and selectively controlled systems. Each method has shown unique advantages, as well as some limitations. Most of the applications of these systems are in the biomedical sector. However, further developments are required to apply these microrobots for clinical applications.

## Figures and Tables

**Table 1:** Key features of different shape forming techniques.

Method	Technique	Key Features	Ref.
Collectively actuated multiple microrobotic systems	Magnetic	Common methods are magnetic torque and magnetic-gradient-based methods. Perform cooperative manipulation tasks.	[40,44,45]
	Acoustic	Simpler low-cost microparticles without specific physical properties. Rapid reversible swarms are formed.	[46,47,48]
	Electric	Requires dielectric particles for actuation. Superimposed DC and AC electric fields are used to improve directional control.	[49,50,51]
	Optical	Spatial selectivity is high. Photothermal or photochemical control methods are significant.	[52,53,54]
Selectively actuated multiple microrobotic systems	Surface anchoring	Anchoring inputs depend on microrobot motion. Surface forces are significant.	[55,56,57]
	Heterogeneous Design	Different geometrical and physical property variations are utilized to perform selective actuation.	[58,59,60]
	Non-uniform control input	Superposition of actuation fields generate non-uniform forces on microrobots.	[61,62,63]

**Table 2:** Advantages and challenges of different shape forming techniques.

Method	Technique	Advantages	Challenges	Ref.
Collectively actuated multiple microrobotic systems	Magnetic	Less expensive and less complicated equipment. Non-contact long-distance action capability.	Programming microrobot motion with underactuated control signals. Low force bearing capacity during micromanipulation.	[40,52,67]
	Acoustic	Fast response. Able to manipulate heavy payloads.	Heat generation during actuation.	[20,74,99]
	Electric	Low-cost setup. Fast assembly of swarms.	Effect of field becomes weaker with distance.	[50,100,101]
	Optical	Availability and low cost.	Inability to penetrate through non-transparent media.	[52,102,103]
Selectively actuated multiple microrobotic systems	Heterogeneous design	External field control is simple.	Precise fabrication and modeling required.	[59,90,91]
	Surface anchoring	Both uniform and non-uniform modules can be used.	Unable to adapt for workspaces far from the control surface.	[57,63]
	Non-uniform input	Better application potential.	Significant requirement of computational effort.	[56,63,89]
